# Hemophilia A and factor V deficiency in a girl with Turner syndrome: a case report

**DOI:** 10.1186/s13256-023-04215-2

**Published:** 2023-11-18

**Authors:** Rawan Al khudari, Duaa Batesh, Roaa Habash, Othman Hamdn

**Affiliations:** 1 Department of Paediatrics, Faculty of Medicine, Damascus University, Damascus, Syria; 2 Faculty of Medicine, Damascus University, Damascus, Syria; 3 Division of Hematology and Oncology, Department of Paediatrics, Faculty of Medicine, Damascus University, Damascus, Syria

**Keywords:** Hemophilia A, Factor V deficiency, Turner syndrome

## Abstract

**Background:**

Hemophilia is an X-linked, recessive inherited disease caused by a defect or deficiency of one of the coagulation factors (VIII or IX). It is considered a rare disease in females. One of the reasons that hemophilia affects females is Turner syndrome. Hemophilia with Turner syndrome is a very rare case, but the combination of Turner syndrome, hemophilia, and factor V deficiency is an isolated case that has never been recorded in the medical literature.

**Case presentation:**

In our case, a 5-year-old Syrian girl presented with hemorrhage of gum, epistaxis, and short stature. The lab tests showed: prolonged activated partial thromboplastin time and prothrombin time with deficiency of factor V (1%) and factor VIII (1%). We diagnosed hemophilia A with factor V deficiency. In addition to short stature, the patient was noted to have spaced nipples and winged neck. We performed karyotyping that showed deletion of one X chromosome (45X0), Turner syndrome. There is no family history of hemophilia or any other genetic disease.

**Conclusions:**

In females affected with hemophilia, karyotyping should be performed. It is very important not to exclude the possibility of a combination of deficiency of more than one clotting factor, and to note that deficiency of more than one factor does not necessarily increase the severity of bleeding compared with deficiency of a single factor.

## Background

Turner syndrome is a multisystem disease caused by deletion of one X chromosome (45X0). It affects females and causes multiple abnormalities (nipples spaced, webbed neck, low hairline) and organ damage (gonadal dysfunction, heart disease, diabetes, liver diseases) [1]. Females with Turner syndrome, albeit rare, could potentially develop X-linked diseases such as hemophilia. Hemophilia is an X-linked, recessive inherited disease caused by deficiency of one of the coagulation factors. Hemophilia has three major types: hemophilia A, B, and C. Hemophilia A and B are the result of deficiency of factor VIII and factor IX, respectively. Hemophilia C, which is rare, is a result of deficiency of clotting factor XI. Hemophilia A affects 1/5000 live male births, while hemophilia B affects 1/30,000 live male births [2]. Hemophilia is rare in females due to the presence of two X chromosomes; however, exceptions include: homozygosity of the hemophilia gene, extreme X-chromosome inactivation in a heterozygote, and a single X chromosome (Turner syndrome) [3, 4].The combined deficiency of coagulation factors V and VIII is a rare autosomal recessive disease with a frequency of 1 in 1,000,000. It is caused by loss-of-function mutations in either *LMAN1* or *MCFD2* [5]. It presents with mild to moderate bleeding tendency and prolonged activated partial thromboplastin time (APTT) and prothrombin time (PT) [6]. Here we report the case of a 5-year-old girl with Turner syndrome who has symptoms of bleeding with short stature, spaced nipples, and winged neck. So far, 13 cases have been recorded in the medical literature as having Turner syndrome with hemophilia, but there is no case that describes the combination of Turner syndrome and factor V (FV) and FVIII deficiency.

## Case presentation

A 5-year-old Syrian girl presented to our clinic with complaint of short stature, hemorrhage of gum, epistaxis, and delayed wound healing.

Her physical examination was normal.

We performed laboratory tests that showed the following: prolonged activated partial thromboplastin time (APTT) (95.0 seconds) and prothrombin time (PT) (21.9 seconds) with normal platelets 375 × 1000/mm^3^, bleeding time (2.40 minutes) and coagulation time (5.10 minutes).

Thus we checked the levels of coagulation factors, we found deficiency of factor V (1%) and factor VIII (1%) with normal values of factors II and X, and von Willebrand factor (vWF). As a result of the symptoms and lab tests, we diagnosed hemophilia A with factor V deficiency.

There was negative family history of bleeding tendency in both paternal and maternal families.

As hemophilia in girls is very rare and the symptoms include short stature, we conducted karyotyping that showed deletion of one X chromosome (45X0). Therefore, Turner syndrome was diagnosed.

We administrate intravenously fresh frozen plasma (FFP) to stop bleeding episodes and Recombinant Factor VIII concentrate. The girl is alive and well. She has a good response to treatment. We referred her to an endocrinologist to treat the short stature.

## Discussion

We presented the case of a 5-year-old girl with Turner syndrome and mild hemophilia A (deficiency of FVIII) with factor V deficiency. There is no family history of hemophilia or any other genetic disease. Her brothers and sisters underwent blood tests, and they were healthy. The clinical symptoms that the patient complained of were hemorrhage of gum, epistaxis, and short stature. The laboratory findings showed combined factor V (FV) and FVIII deficiency (CF5F8D). Hemophilia is an X-linked, recessive inherited disease caused by a defect or deficiency of coagulation factor VIII (hemophilia A) or factor IX (hemophilia B) [7]. Hemophilia is characterized by easy bruising, epistaxis, and bleeding into joints and can occur after trauma or surgery and menorrhagia [8]. Symptoms of hemophilia are classified as mild, moderate, and severe, depending on the normal activity of the clotting factor VIII or IX. If the concentration of factor VIII or IX is more than 5%, it is mild, in moderate hemophilia between 1% and 5%, and in severe hemophilia less than 1%. Generally, diagnosis of moderate or mild hemophilia may be delayed until adulthood whereas severe hemophilia appears in infants [7]. This disorder manifests mainly in males, whereas it is considered a rare disease in females. Typically, females are carriers as they are homozygous. The potential causes that could lead to affected female are skewed lyonization: skewed inactivation of one X chromosome, co-inheritance of mutations from both parents (affected father and carrier mother), and hemizygosity (a single X chromosome, Turner’s syndrome) [4]. Therefore, the laboratory findings showed deficiency of factors V and VIII in a female patient combined with a short stature complaint, and we noted spaced nipples and winged neck, prompting us to perform karyotyping, which showed deletion of one X chromosome (45X0), Turner syndrome. Turner syndrome is a genetic disorder caused by the deletion of one X chromosome [1]. The symptoms and clinical features are variable, depending on the extent of X-chromosome deletion. The symptoms are very intensified, and the diagnosis is made in the prenatal period if the entire X chromosome is missing, whereas if the deleted amount is small the first symptom may be primary amenorrhea and inability to get pregnant [9]. Clinical manifestations of Turner syndrome include short stature, neck webbing, swelling, broad chest with spaced nipples, low posterior hairline, low-set ears, and hand edema. In addition to organs disorders (gonadal dysfunction, infertility, heart disease, diabetes, liver diseases, and increased risk of autoimmune disease) [10]. It is important to pay attention to clinical manifestations of Turner syndrome. It may not be obvious in the patient, which leads to a delay of diagnosis [9]. In our patient, the parents only complained of short stature, but we observed spaced nipples and winged neck. The treatment of Turner syndrome varies depending on the period of life. We focus on growth during childhood and adolescence, so we use growth hormone (GH) [11]. Studies showed the benefit of using GH by increasing the length by about 7–15 cm [12], but this increase does not appear in every girl with Turner syndrome. It depends on several factors, including time of diagnosis, time of therapy, and unsuitable dose [13]. Thirteen cases have been recorded in the medical literature having Turner syndrome with hemophilia, but there is no case that describes the combination between Turner syndrome and factor V (FV) and FVIII deficiency. Combined deficiency of coagulation factors V and VIII (F5F8D) is an autosomal recessive coagulation disorder caused by mutations in *LMAN1* or *MCFD2 * [5]. The function of *LMAN1* and *MCFD2* as a cargo receptor is essential for effective secretion of coagulation factors V (FV) and VIII (FVIII) [14, 15]. F5F8D is a rare disorder with a frequency of 1 in 1,000,000 [5]. It is characterized by mild to moderate spontaneous bleeding manifestations such as bleeding of gum, epistaxis, easy bruising, and menorrhagia in females, in addition to bleeding into joints, gastrointestinal bleeding, and intracranial bleeding, which are uncommon symptoms [16]. The combination of factors VIII and FV deficiency does not increase the severity of bleeding compared with deficiency of a single factor [17]. We are giving the patient fresh frozen plasma (which contains factors V and VIII) and factor VIII concentrate (which contains only factor VIII), and we referred the patient to an endocrinologist to treat the short stature.

## Conclusion

Hemophilia is a rare disease in females, who are usually only carriers. Hemophilia can affect females who have a single X chromosome (Turner syndrome) in rare cases. We present the first case report that combines Turner syndrome with (FV) and (FVIII) deficiency. Karyotyping is necessary in females with hemophilia to determine the cause, as Turner syndrome may have no obvious manifestations.

## Data Availability

Any additional data or material is available on request.

## Abbreviations

APTT: Activated partial thromboplastin time
PT: Prothrombin time
vWF: Von Willebrand factor
CF5F8D: Combined factor V (FV) and FVIII deficiency
